# Anti-pandemic restrictions, uncertainty and sentiment in seven countries

**DOI:** 10.1007/s10644-022-09447-8

**Published:** 2022-10-09

**Authors:** Wojciech Charemza, Svetlana Makarova, Krzysztof Rybiński

**Affiliations:** 1 Vistula University, Warsaw, Poland; 2 University of Leicester, Leicester, UK; 3 University College London, London, UK

**Keywords:** Anti-pandemic government policy, Newspaper-based uncertainty measure, Country effects, Machine learning, F520, H12, I18, D83, D81, Z18

## Abstract

We investigate how the stringency of government anti-pandemic policy measures might affect economic policy uncertainty in countries with different degrees of press freedom, various press reporting styles and writing conventions. We apply a text-based measure of uncertainty using data from over 400,000 press articles from Belarus, Kazakhstan, Poland, Russia, Ukraine, the UK and the USA published before the wide-scale vaccination programmes were introduced. The measure accounts for pandemic-related words and negative sentiment scores weight the selected articles. We then tested the dynamic panel data model where the relative changes in these measures were explained by levels and changes in the stringency measures. We have found that introducing and then maintaining unchanged for a relatively long time a constant level of anti-pandemic stringency measures reduce uncertainty. In contrast, a change in such a level has the opposite effect. This result is robust across the countries, despite their differences in political systems, press control and freedom of speech.

## Introduction

This paper investigates the relationship between anti-pandemic restrictions and policy-related uncertainty. It is already well-known that anti-pandemic measures like lockdowns, limits on social contacts and travel restrictions have had numerous negative economic and social effects (see, e.g. Arnon et al. 2020; Coibion et al. 2020; Brodeur et al. 2021; Baig et al. 2021; Mdaghri et al. 2021; Berman et al. 2021; and many others). However, the question of how anti-pandemic measures, or government intervention more generally, are related to policy uncertainty is still open. It is known that such uncertainty has a predominantly negative impact on economic growth (see the seminal Bloom et al. 2018; and numerous other empirical papers). There is also evidence (see, e.g. Liu et al. 2022) of the negative impact of the pandemic restriction-caused uncertainty on the real and financial sectors of the economy.

In this study, we look at the problem of to what extent imposing and maintaining anti-pandemic restrictions affects uncertainty. It is widely accepted that imposing new restrictions on the movements of people and goods, particularly at short notice, increases all forms of uncertainty. However, such restrictions are imposed, and obeyed differently in different countries, so they might affect uncertainty differently. It is often pointed out that reducing these elements of policy uncertainty, which are, to an extent, controlled by policy-makers, can benefit the economy (see McMahon 2019). The relations between policy uncertainty and policy action evidently become more relevant at times when such interventions are frequent and drastic, like during the pandemic. These relations might also depend on country-specific factors like the degree of administrative centralisation, social discipline, demography, industrial structures, the penalty system or the freedom of media and speech.

Not surprisingly, the evidence shows that economic uncertainty in its various forms, which had already been at a historically high level before 2020, increased further during the first waves of the Covid-19 pandemic in 2020 (see Altig et al. 2020; Barrero and Bloom 2020; Meyer et al. 2021 and many others). The increase was observed worldwide and is well-documented by the uncertainty and volatility measures such as the Infectious Disease EMV Tracker (Baker et al. 2020a) and the World Pandemic Uncertainty Index (WUPI) (see Ahir et al. (2021)). Other studies examine how the additional uncertainty generated in stressful times like pandemics affects the financial and non-financial world (see Nalban and Smădu 2021; Zhang Y and Hamori 2021; Bahmani‑Oskooee and Xu 2022; Ongan and Gocer 2022; and, less directly, Kim 2021). Even so, the studies of various types of economic uncertainty during the pandemic have not specifically analysed how it is related to the strength, dynamics and magnitude of the anti-pandemic measures taken by governments in a country-specific context.

We focus on how the anti-pandemic restrictions imposed by governments have affected policy-related uncertainty, and we look at this from a cross-country and multi-lingual perspective. Faced with a plethora of different ways of measuring uncertainty (see, e.g. Mumtaz and Teodoridis 2017; Redl 2020; and Apaitan et al. 2022; for comparison of the cross-country and country-specific approaches), we limit our interest to text-based measures of uncertainty. These measures were first proposed by Baker et al. (2016) in the form of the economic policy uncertainty (EPU) index. This is constructed by searching newspapers for words with an economic sense that appear in conjunction with words describing policy and uncertainty. The frequency with which such words appear in the press is the base for the EPU measure of uncertainty. The studies of pandemic-related uncertainty published so far have predominantly looked at English-speaking sources of textual data, and the largest, in its coverage of the WUPI index, is based on texts in English. As reporting styles for news might differ significantly between languages (see Thomson 2008), relying on English-language sources to assess uncertainty might give results that are of limited relevance. Such an index might be very useful for assessing how uncertainty about a given country is reported internationally. Still, the perception of uncertainty within a country might be quite different, particularly if that country is, to a degree, politically or socially isolated from an English-language environment. To analyse whether country-specific factors influence the effects of the pandemic-related restrictions on the text-based measures of uncertainty, we focus on countries that vary substantially in the social and political aspects of their information and press policy. We also consider countries where language and social diversification mean that the contents of reports on the pandemic differ in their tones and emphasis.

To account for some of the differences in how different languages describe uncertainty, we consider the sentiments expressed in the articles analysed. These differences might be substantial. The results of Jha et al. (2021) show that the magnitude of the expressions of sentiment towards finance in books published in Chinese, English (UK and US separately), French, German, Italian, Russian and Spanish in the period 1870–2009 is markedly different for each of these languages, with the lowest sentiment score recorded for Russian. These differences might be particularly relevant where they are found in reporting on earlier pandemic crises, and health scares when factual information was often given with negative and sometimes hysterical undertones (see, e.g. Clarke and Everest 2006; Ribeiro et al. 2018). The same has been observed during the current pandemic (see, e.g. Bagus et al. 2021).

We conjecture from these findings, though we do not test it in this paper that the cascade of information in the press about the pandemic might crowd out other news because the press has only a limited capacity. This might reduce the amount of economic and policy information that is given. This could cause bias in the traditionally computed EPU-style indices, as there might be relatively fewer articles that can be classified as containing economic and political words in a context other than the pandemic. Additionally, the words or descriptors traditionally used to explain uncertainty might be replaced by more medically or epidemiologically oriented terms. This might also add to the bias. Finally, the differences in how languages express sentiments might also be a factor here. We modify the original EPU methodology to construct pandemic-related uncertainty indices to deal with these problems. The methodology differs from the EPU methodology in two main aspects. First, we add pandemic-related words to the descriptors, roughly similar to how Barrero and Bloom (2020) computed their health-augmented EPU index for the US. The difference is seen in the selection of pandemic-related words and the choice of data sources. The second difference is that we weigh the newspaper articles by the frequencies obtained from the negative sentiment scores, so we assume that an increase in the density of words associated with negative sentiment increases the uncertainty. To check the robustness of our approach, we then construct a series of different uncertainty indices for each country with and without these additional elements and with sentiments measured in different ways. Next, we estimate a dynamic panel data model, where our uncertainty indices are explained by factors related to the pandemic and a measure of the stringency of each government’s anti-pandemic policy. We estimate the model using weekly data from 2 March 2020 to 21 March 2021, covering the first two waves of the pandemic in Belarus, Russia, Poland and Ukraine, and three waves in the UK and the USA; waves are more difficult to identify for Kazakhstan. We decided not to use data from later periods, as vaccination programmes intensified in Spring 2021 in most of the countries in the panel, causing structural changes. Such changes call for a different technique to be applied, and we leave the analysis for further studies.

In the absence of an appropriate theory, we resort to formulating a simple hypothesis that levels of restrictions and changes in them might affect the dynamics of uncertainty, and we test this by looking at the data. We construct a panel of weekly text-based uncertainty indices for Belarus, Kazakhstan, Poland, Russia, Ukraine, the UK and the USA, building it from data from newspapers in the local language. We deliberately selected countries where the degree of media freedom, journalistic styles and conventions, language features, press perception and readership are vastly different, allowing for some idiosyncratic effects. The countries in the panel have applied markedly different anti-pandemic measures and policies, with the most relaxed in Belarus and the most severe in Kazakhstan. In this situation, it becomes interesting and challenging to assess whether the effects of the anti-pandemic measures on uncertainty have followed a similar pattern.

The plan of the paper is as follows. Section 2 describes the data, the way our health and sentiment-weighted uncertainty indices are computed and the measure of the pandemic response of governments. Section 3 discusses the model settings, their limitations and particular variants, the estimation results and their robustness. Section 4 concludes.

1. the data: pandemic-augmented uncertainty measures and stringency trackers

The EPU-style indices are essentially constructed as follows (see Baker et al. 2016):

1. Define descriptors that are sets of words and phrases that characterise ‘economic’, ‘policy’ and ‘uncertainty’.
2. Do a machine search of all the articles in the database to identify those containing at least one word from each set of descriptors.
3. Aggregate and scale the selected articles and construct an index reflecting the frequency of the EPU-related newspaper articles.

In Sect. 1, we argue that the EPU-style newspaper-based uncertainty measures constructed in this way may not fully reflect the substantial increase in uncertainty during the pandemic. Consequently, we augment the set of ‘uncertainty’ descriptors to account for words related to the pandemic like ‘covid’ and ‘coronavirus’. The words we added to the descriptors translate into English as ‘virus’, ‘viral’, ‘infect’, ‘coronavirus’, ‘covid’, ‘pandemic’ and ‘epidemic’. This approach is, to an extent, similar to that of Zhang W. and Hamori 2021. It should be noted that other pandemic-related text-based measures and trackers, e.g. Infectious Disease EMV Tracker and WUPI, use slightly different sets of pandemic-related words. Our choice is related mainly to the fact that the words we use are incorporated or easily translated into the languages of the non-English countries investigated. A direct translation of the original descriptors might cause the relevant context to be lost or distorted because of linguistic diversity. This diversity might in certain languages result in some words from the sets of descriptors being avoided for social or political reasons and substituted by words that are more, or sometimes less, directly related to the intended message, leading to bias. A typical example might be the fuzzy communication content of the noun ‘uncertainty’, which might easily be lost in direct or indirect translation (for a discussion of the problems in understanding it in English, see, e.g. Babrow 2001 and Angelone 2010).

Consequently, we have decided to weight the index by the sentiment score of each selected article. We use the lexicon-based approach, as in Taboada et al. (2011). That is, each article selected as containing the desired descriptors is searched for words associated with negative sentiments like ‘bad’, ‘disastrous’, ‘gloomy’ or similar. For each language, collections of such words are available as sentiment lexicons. For the Russian-language press, we apply the RuSentiLex lexicon by Loukachevitch and Levchik (2016), accessible at https://www.labinform.ru/pub/rusentilex/, which contains about 12,000 words. The English-language lexicon is at https://www.cs.uic.edu/~liub/FBS/sentiment-analysis.html#lexicon, and the Polish lexicon is described in Zaśko-Zielińska et al. (2015). The lexicon-based approach is often regarded as inferior to that based on the machine learning, particularly BERT (see Devlin et al. 2019). However, the paper by Kotelnikova et al. (2021) shows that a simple lexicon-based approach often gives comparable, if not better, results than BERT. The details of the methodology of weighting the uncertainty scores by sentiments are given in Appendix A.

The analysis was made using a panel of weekly media data from 2 March 2020 to 21 March 2021. For Belarus, Kazakhstan and Ukraine, which are countries with a substantial, and often prevailing, Russian-language press, we choose only newspapers published in Russian. The media sources we use are:

Belarus:3 newspapers: Sovetskaya Belorussiya (SB), Delovaya Gazeta (DG) and BelGazeta (BG)

Kazakhstan:3 newspapers: *Informburo.kz* (IB), *Tengrinews* (TN) and *Zakon.kz* (ZK)

Poland: 3 newspapers: Gazeta Wyborcza (GW), Rzeczpospolita (RZ) and W Polityce (WP)

Russia:4 newspapers: *Izvestiya* (IZ), *Kommersant* (KM), *Novaya Gazeta* (NG) and *Vedomosti* (VD)

Ukraine:3 newspapers: *KP v Ukraine* (KP), *Segodnya* (SG) and *Vesti-UA* (VE)

UK:1 newspaper: *The Guardian* (G)

US:1 newspaper: *The New York Times* (NYT)

The total number of press articles searched is over 430,000 articles, with the majority of articles from the Russian-language press of Belarus, Kazakhstan and Russia. The reason for this imbalance between countries in the number of articles searched is that we intend to concentrate on the effects of governmental anti-pandemic policy in non-English-speaking countries and use the English-language press for comparison. How well *The Guardian* and *The New York Times* represent the English-language press might be debatable, as there are evident differences in the reporting styles and emotional slant of different newspapers (see, e.g. Fu and Dhonnchadha 2020). How this might affect our results is discussed further in this section.

The extent of press freedom and the constraints on reporting varies widely across the countries in the panel, and the journals selected reasonably represent different political orientations and social interests. The 2021 World Press Freedom Ranking (https://rsf.org/en/ranking) puts the UK and the USA in the first quartile on the list of countries for the freedom of the press ranked from best to worst, with the USA on the borderline between the first and second quartiles. Poland is in the second quartile, Ukraine in the third, and Belarus and Russia in the fourth. After invading Ukraine in February 2022, the media freedom in Russia effectively ended, but this is outside the period we investigated. Nevertheless, the distributions of words selected here as descriptors of uncertainty and sentiment are generally similar across the countries and journals. This is illustrated by Table 1, which shows basic descriptive characteristics of the distributions of the unscaled frequencies of negative sentiments for all seven countries. These results indicate that the bias in reporting uncertainty and news about the pandemic is not substantial and should not distort the results.

**Table 1:** Descriptive characteristics of the distributions of the negative sentiments in all the newspapers analysed

	Belarus^(1)^			Kazakhstan^(2)^		
	SB	DG	BG	IB	TN	ZK
No.obs	16,608	10,899	39,738	13,848	20,316	57,098
Mean	0.055	0.069	0.057	0.054	0.064	0.065
Median	0.048	0.067	0.050	0.045	0.056	0.056
st.dev	0.037	0.026	0.033	0.044	0.044	0.045
interq. R	0.046	0.030	0.042	0.055	0.059	0.058
Skewness	0.541	0.253	0.623	0.620	0.545	0.560

	Poland^(3)^			Russia^(4)^			
	GW	RZ	WP	IZ	KM	NG	VD
No.obs	32,093	25,392	25,698	20,480	24,872	3151	13,478
Mean	0.118	0.121	0.135	0.069	0.060	0.076	0.054
Median	0.112	0.113	0.128	0.065	0.056	0.072	0.047
st.dev	0.059	0.067	0.063	0.036	0.031	0.031	0.036
interq. R	0.075	0.078	0.082	0.048	0.039	0.037	0.041
Skewness	0.316	0.358	0.322	0.335	0.425	0.392	0.587

	Ukraine^(5)^			UK^(6)^	US^(7)^
	KP	SG	VE	G	NYT
No.obs	105,687	7813	20,190	14,194	68,799
Mean	0.064	0.064	0.061	0.061	0.056
Median	0.058	0.057	0.057	0.059	0.054
st.dev	0.036	0.037	0.031	0.024	0.024
interq. R	0.047	0.050	0.037	0.032	0.031
Skewness	0.484	0.589	0.449	0.251	0.193

*No.obs* number of observations, *st.dev.* standard deviation, *inerq.r* interquartile range

1. SB: Sovetskaya Belorussiya; DG: Delovaya Gazeta; BG: BelGazeta;

2. IB: Informburo.kz; TN: Tengrinews; ZK: Zakon.kz.

3. GW: Gazeta Wyborcza; RZ: Rzeczpospolita; WP: W Polityce.

4. IZ: Izvestiya; KM: Kommersant; NG: Novaya Gazeta; VD: Vedomosti.

5. KP: KP v Ukraine; SG: Segodnya; VE: Vesti-UA.

6. G: The Guardian.

7. NYT: The New York Times.

We made an additional check to find out for Belarus, a country with a heavily controlled press and a government-induced bias towards reporting international events, whether words related to the pandemic appear in articles that might be identified as relating to international than domestic affairs. If the pandemic is associated with international news, our measure of uncertainty might be biased, as it would reflect reporting on the pandemic abroad rather than on the domestic situation. For this check, we conduct machine learning-based topic modelling. We apply the unsupervised latent Dirichlet allocation algorithm (see, e.g. Blei et al. 2003) to identify the leading topics of the articles in the Belarusian press that contain at least one word from each set of descriptors. Using models with the number of pre-assessed topics for each article equal to 5, 10 and 15, we find that none of these topics can be clearly identified as directly related to international or foreign affairs. Consequently, we conclude that the Belarusian press articles with pandemic-related words concern the domestic pandemic rather than worldwide events. That is, they are consistent with the indices for the other countries considered here. Similar checks have been done for Russia, with results published in Charemza et al. (2022).

Due to a lack of available data, we are not able to analyse uncertainty and sentiments expressed in the newspapers published in Ukraine and Kazakhstan in their native languages rather than in Russian. This might cause some bias in our results, particularly for Ukraine, where the political and social differences between the Russian- and Ukrainian-speaking regions might be significant. This problem might be of minor importance for Belarus as the tight press control results in publication in the Belarussian language press economic and general interest articles, essentially duplicating the Russian-language articles.

**Fig. 1:**
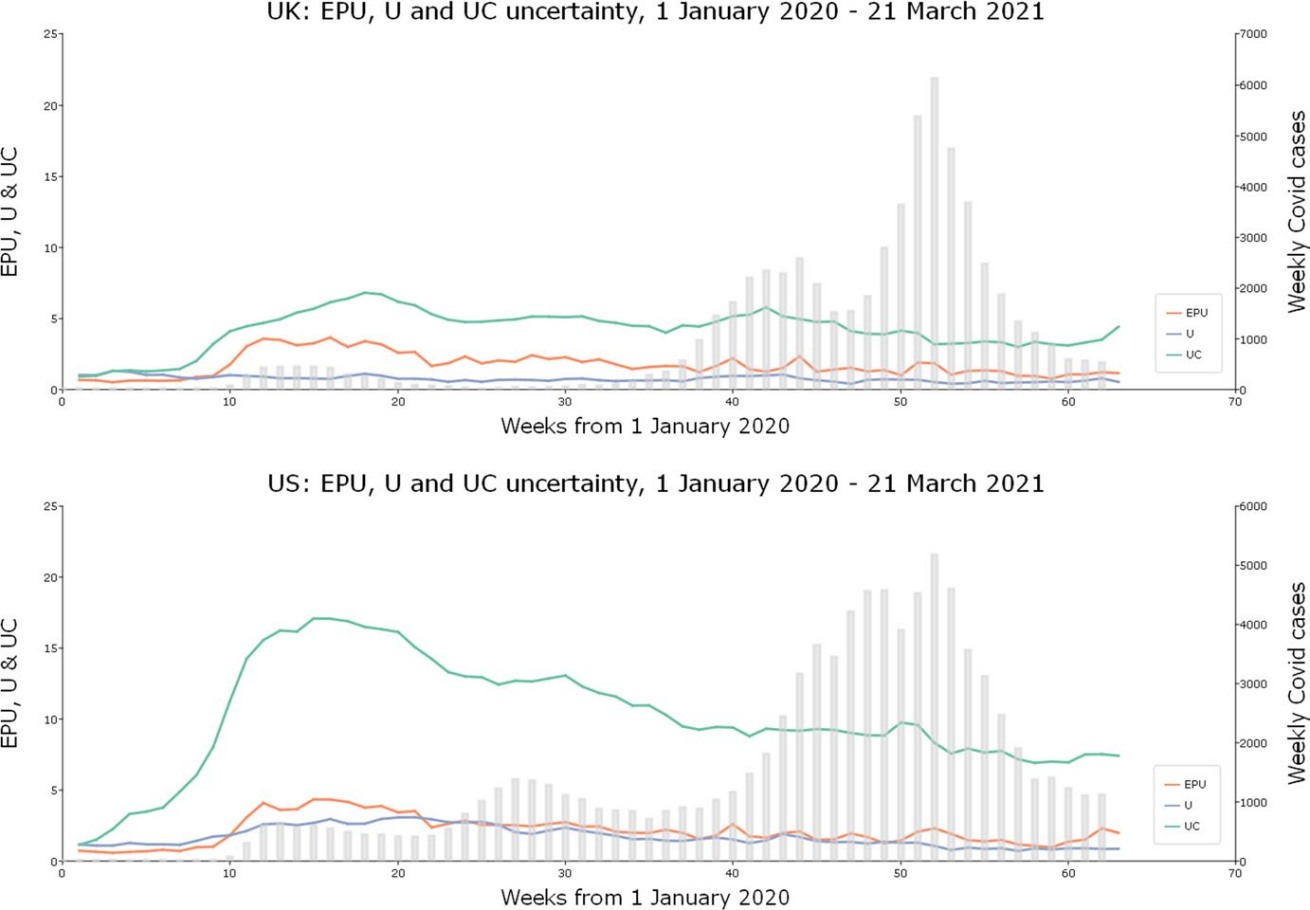
Uncertainty indices, with and without pandemic-related and sentiment augmentations. *Legend*: EPU: original EPU index, recomputed from daily data available at https://www.policyuncertainty.com/us_monthly.html; U: uncertainty index, constructed without pandemic and sentiment-related component using the methodology consistent with EPU methodology; UC: pandemic-augmented and sentiment-weighted uncertainty index. Data for Covid-19 cases: https://github.com/owid/covid-19-data/blob/master/public/data/

Figure 1 illustrates the impact of including pandemic-related words in constructing the uncertainty index. We compare our uncertainty pandemic and sentiment-weighted indices for the UK and the USA with the original EPU indices computed from the daily data available on the EPU website https://www.policyuncertainty.com/ us_monthly.html. The website also shows the unfolding of the pandemic measured by newly registered cases of Covid-19 each week. The EPU indices are constructed using data from about 650 newspapers for the UK and over 1,000 for the USA, while our indices are computed using only one newspaper for each of these countries, taking *The Guardian* for the UK and *The New York Times* for the USA. For comparison, all the indices are scaled to 1 for the first week of 2020. Our index, constructed without the pandemic and sentiment-related components, is consistent with the original EPU methodology and is denoted as ${\text{U}}$, and our pandemic-augmented and negative sentiment-weighted index is UC, with C for ‘coronavirus’. It should be noted that Zhang and Hamori (2021a and 2021b) also applied sentiment lexicon-based measures for the analysis of uncertainty.

For the USA, the dynamics of the EPU and U indices are very similar, even though U is made using data from only one newspaper. This suggests that the heterogeneity of reporting styles does not affect the uncertainty estimates for the USA. This is, however, not the case for the UC index, which clearly dominates over the non-pandemic-augmented indices. This illustrates the effect of the pandemic-related news in crowding out other types of information. For the UK, the EPU index is somewhere between the original EPU and the augmented index, which might reflect the different ways the EPU indices are constructed for the two countries.

In the period under investigation, the countries selected took vastly different approaches to the pandemic and to imposing anti-pandemic restrictions. We may summarise here the main points of these policies for each country (for more information, see, e.g. the World Health Organisation’s Covid-19 Health System Response Monitor (https://eurohealthobservatory.who.int/monitors/hsrm/overview).

In Belarus, reasonably light restrictions on the self-isolation of people travelling to the country were introduced in March 2020. They were subsequently strengthened in April and then remained virtually unchanged until early October, when they were strengthened further, extended and clarified. However, the Belarusian official data on the pandemic are expected to be biased and not very accurate (see Nemira et al. 2021).

Kazakhstan introduced severe anti-pandemic restrictions in May 2020. These restrictions were, however, limited to the main cities, where, occasionally, whole quarters were isolated and cordoned off when Covid-19 outbreaks were identified. However, quarantine measures for the entire country were only introduced in early June 2020 and then relaxed slightly in December 2020. The reported dynamics of the pandemic in Kazakhstan is different to those in the other countries in the panel, possibly because of the paucity of data (see Yegorov et al. 2021).

In Poland, the anti-pandemic restrictions were changed more frequently than elsewhere during the period investigated, and they varied in scope and severity. Schools were closed several times and then opened again. Overall, the lack of clarity created some confusion and misunderstandings about the rules and restrictions. There is also some evidence of censorship of information about the pandemic, particularly in 2020 (see Abazi 2020; Speier 2021).

In Russia, restrictions varied markedly between regions and cities but were generally not very severe. However, Russia imposed administrative and penal liability for disseminating false information about the pandemic, which might have affected the reporting by the press to some extent (see, e.g. Yadav et al. 2021). The data on deaths and cases of Covid-19 might be flawed as well (see Dyer 2020).

In Ukraine, the restrictions were reasonably light until September 2020, with an emphasis on information policy rather than restrictions on movement. More severe restrictions on self-isolation and high-risk areas were introduced in September and October 2020. However, reservations were raised about the disinformation campaign of the pandemic in Ukraine, which might affect data quality and media coverage (see Patel et al. 2020).

In the United Kingdom, restrictions varied between England, Scotland, Wales, Northern Ireland, and individual cities and counties. They have generally been in line with the data on the dynamics of the pandemic. In the USA, the types and severity of restrictions were different in individual states and were quite light compared to those in other countries and often not compulsory. The differences were polarised further by the widespread resistance to the anti-pandemic measures. There are also reservations about the bias in media coverage of the pandemic in the UK and the USA (e.g. Yang et al. 2021; Zhao et al. 2020).

Because of the diversity of the methods used to tackle the pandemic crisis, aggregate measurement of government policies would inevitably be controversial and complicated. For our study, we turn to the measures known as the Coronavirus Government Response Tracker, OxCGRT; see Hale et al. (2021). The OxCGRT aggregates information about the various types of pandemic-related restrictions and their severity. This gives a collection of indices, each of which is constructed with different selection restrictions imposed by the government. Our interest is centred around four of these indices:

1. *The Stringency Index* (SI). This is the basic index computed using data on nine main components: school closures, workplace closures, cancellation of public events, restrictions on gatherings, stoppages of public transport, stay-at-home requirements, restrictions on internal movement, restrictions on international travel and public information campaigns.
2. *The Legacy Stringency Index* (LSI). This is the earlier version of the Stringency Index. It differs from the Stringency Index by omitting restrictions on gathering and stay-at-home requirements.
3. *The Containment and Health Index* (CHI). This is an extended version of the Stringency Index that includes testing policy, contact tracing, facial covering requirements and vaccination policy data.
4. *The Government Response Index* (GRI). This is the fullest version of the index, as it adds data on income support and debt or contract relief for households on top of the CHI components.

All these indices have been widely used in a number of studies on how government restrictions have impacted various economic, political, social and health phenomena (see, e.g. Bargain and Aminjonov 2020; Pulejo and Querubin 2021; for a partial review, see Hale et al. 2021). There are also other measures of anti-pandemic policy stringency and indices developed using different criteria and data (see, e.g. Gros et al. 2021 for a description of the measures developed for European countries; and Cot et al. 2021 for the application of Google Mobility Data). We concentrate on the fullest version of the OxCGRT tracker, the Government Response Index, GR, using the other tracker versions in our robustness analysis.

Although daily records are available, we decide to use weekly rather than daily data. The Covid-related daily data contain excessive noise as they are often recorded and released in some weekly patterns. This would create large numbers of missing observations and weekly cyclicality that is different for each country in the panel. Also, the daily newspapers express a sort of weekly cyclicality publishing specific information on particular days of the week. To avoid computational and interpretational problems and to keep the dynamics of the modelled process reasonably simple, we aggregate the daily data into weeks.

**Fig. 2:**
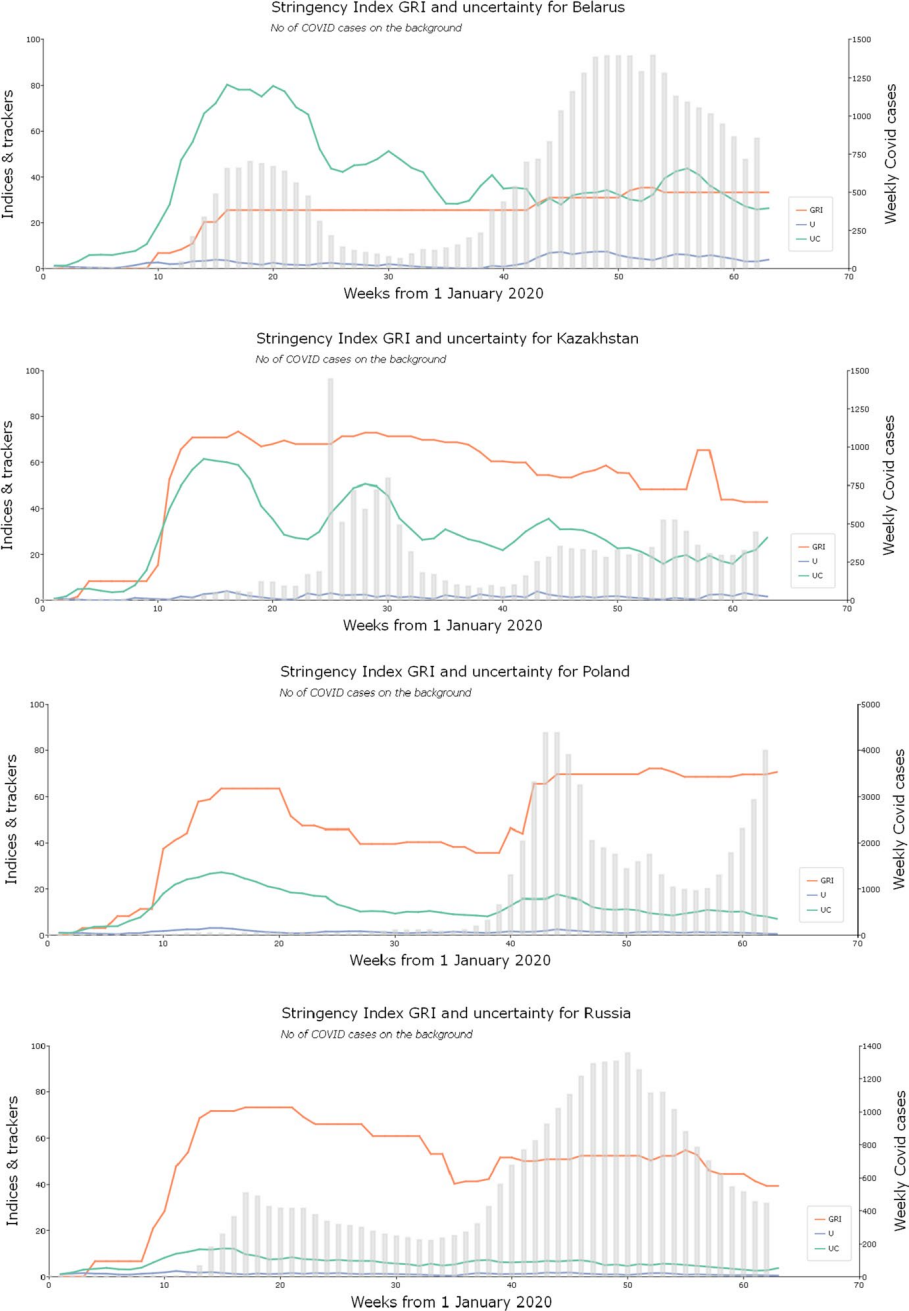

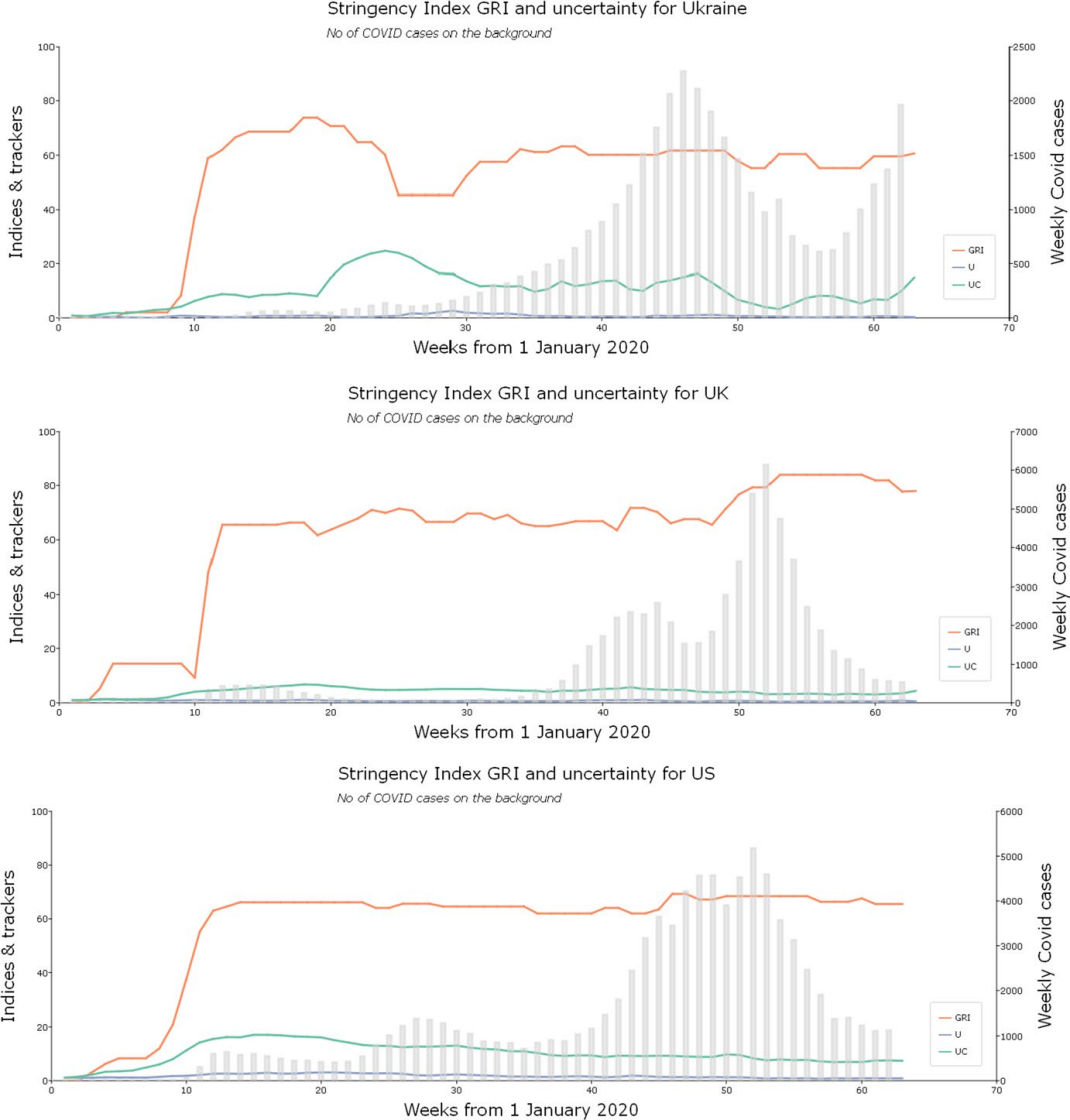
Development of the pandemic, the policy stringency measures and the uncertainty indices. *Legend*: GRI: The Government Response Index; EPU: Economic Policy uncertainty index; U: uncertainty index, constructed without pandemic and sentiment-related component using the methodology consistent with EPU methodology; UC: pandemic-augmented and sentiment-weighted uncertainty index

Figure 2 visualises the weekly data. It compares the U and UC indices for each country in the panel and shows the development of the OxCGRT measures in their fullest GRI version. It also illustrates in the background of each graph the progress of the pandemic using a histogram depicting newly registered cases of Covid-19 each week. The U and UC indices are scaled by the highest value of the dominant index, which is always UC, which means the uncertainty indices are comparable for each country but not comparable between countries. The GRI indices are not scaled, so they are comparable between countries.

Figure 2 reveals some interesting insights into how particular countries reacted to the pandemic news through changing uncertainty and government policy. The pattern is the same for all the countries as the EPU-type uncertainty U and pandemic and sentiment-weighted uncertainty UC are related, but the augmented and sentiment-weighted indices are markedly higher than the corresponding not-augmented indices. The general dynamics of the uncertainty in relation to the news about Covid-19 are also similar, as the peak in uncertainty corresponds to the initial stages of the pandemic in all the countries in the panel. The uncertainty fell slowly after March and April 2020, though it remained at a high level. However, the relationship between the hard and factual news about the pandemic expressed as the number of Covid-19 cases discovered, the government response, and the reaction of uncertainty are less homogeneous. The faster and strongest pre-emptive reactions to the news about the pandemic were in Kazakhstan, Poland and Ukraine, shown by the prompt imposition of restrictive policy measures ahead of a visible increase in Covid-19 cases. Such a pre-emptive policy appears effective, as the spring 2020 wave of the coronavirus was barely visible in these countries. Other countries reacted slightly more slowly. The longest delay of an increase in uncertainty in relation to the imposition of government restrictions was in Ukraine. The governments of Russia, the US, the UK and particularly Belarus did not take any pre-emptive action in imposing the restrictions. They only reacted after the effects of the pandemic were already clearly evident within their countries.

## Results, robustness and discussion

Given that the evidence presented in Sect. 2 about the relationship between uncertainty and government policy restrictions shows some repeating patterns, we extend our analysis by attempting to quantify this relationship in a panel data model. We base our analysis on the following dynamic model:

1 $$\begin{gathered} y_{it} = \mu_{i} + \theta_{1} (L)g_{it} + \theta_{2} (L)\Delta g_{it} + \theta_{3} (L)x_{it} + \theta_{4} (L){cross}_{it} + u_{it} , \hfill \\ L^{ * } (u_{it} ) = \varepsilon_{t} \hfill \\ \end{gathered}$$

where $y_{it}$ denotes the logarithms of the weekly changes in the uncertainty index for country $i$ in week $t$, $\mu_{i}$ is country-specific fixed effects, $g_{it}$ is the logarithm of one of the OxCGRT government anti-pandemic stringency trackers described in Sect. 2, $\Delta g_{it}$ is the rate of change in the tracker, $x_{it}$ stands for other external factors related to the pandemic, $cross_{it}$ denotes the interaction effects, which are the products of the selected $g_{it}$, $\Delta g_{it}$ or $x_{it}$ variable with the dummy variable indicating the $i$ th country, $u_{it}$ is a possibly weakly dependent error term such that $\varepsilon_{it} \sim iid(0\;,\;\sigma_{\varepsilon }^{2} )$, $L^{j}$ is lag operator of order $j$, and $L$ is the polynomial lag operator such that $\theta_{k} (L) = \mathop \sum \limits_{j = 1}^{n} \theta_{k,j} L^{j}$, $L^{ * } = 1 - \mathop \sum \limits_{j = 1}^{n} \rho_{j} L^{j} \;\;$. As we use logarithms, we rescale the original variables containing zeros by shifting them by one unit so that ones replace the zeros.

We treat a change in restrictions as a shock, even if it has been pre-announced. It is justified by the unprecedented nature of the pandemic and the resulting lack of experience in adjusting to new situations. Consequently, we expect that the cumulative effect of changes in restrictions is positive, that is, $\mathop \sum \limits_{j = 1}^{n} \theta_{2,j} > 0$, which means that an increase in the stringency of the anti-pandemic measures leads to an increase in uncertainty. The total effect of the severity of the restrictions on uncertainty is, however, less clear. It depends on the nature of the restrictions. Generally, if there is a degree of trust and confidence in governments’ actions, more severe restrictions should provide reassurance that the pandemic situation is dealt with, reducing uncertainty. On the other hand, restrictions on hospital admissions for non-Covid patients might have a long-lasting effect, for instance. Hence, we cannot hypothesise on the expected sign of $\mathop \sum \limits_{j = 1}^{n} \theta_{1,j}$, as it depends on the balance of the gradually diminishing effect of the shock caused by introducing a new restriction and the possibly stabilising effect of lasting restrictions.

The principal problem with estimating model (1) is the non-trivial dynamics of the pandemic process and the related indicators, as these affect the statistical properties of the data and, consequently the quality of the estimation. The development of the pandemic and consequently the coverage of it by the press has been of an explosive nature, which theoretical models of the pandemic widely confirm (see, e.g. Eichenbaum et al. 2021), and it has been reported by observation of the pandemic reproduction factor, known as the R factor. Under these conditions, the usual assumptions of stationarity and normality might not be valid. Also, testing for the panel data unit roots might not be appropriate. Firstly, the intensification of the vaccination programmes caused a significant structural break after the end of the sample period, so the asymptotics of such tests might not be valid. Secondly, even if it was valid, the explosive nature of the pandemic spread process would have also invalidated such tests, as in the case of other investigated explosive processes, e.g. financial speculative bubbles. However, the results of Phillips and Magdalinos (2007) and Tao and Yu (2020) for models with similar stochastic properties are mildly encouraging. These results confirm that the OLS-based estimation methods might give parameter estimates with admissible statistical properties. Their findings show, however, that such models might be awkward to test statistically. Moreover, it should be noted that the regressors are weakly rather than strongly exogenous, as there may be dynamic feedback from the uncertainty to the anti-pandemic measures. It might result in the appearance of the reversed causality problem. This does not affect the consistency of the estimators but might affect their efficiency and, in small samples, result in bias of the estimates. It is also assumed that there are no non-random factors other than those related to the pandemic and captured by the data that might affect uncertainty in a systematic way. Following on from these general findings, we decide to resort to the OLS-based estimations with the following precautions:

1. We attempt to apply the Han et al. (2014) approach of using the X-difference technique to estimate the autocorrelation part of the model. This approach is based on fairly weak stochastic conditions. In particular, this technique deals, at least to an extent, with the dynamic feedback problem. As we were not very successful with it (see below in this section), we also applied the different estimators referenced below.
2. We resort to bootstrap inference in testing rather than using analytical tests.
3. We pay particular attention to the robustness of the results obtained by the different estimation techniques.

In reducing model (1), we apply the general-to-specific approach (see, e.g. Campos et al. 2005) combined with a Cochrane and Orcutt (1949) algorithm to eliminate autocorrelation. We start from a possibly general model, meaning one with long lags, using dynamic fixed-effects OLS and initially estimating the first-order autocorrelation coefficient with one of the methods listed further on. Suppose there is significant autocorrelation in the residuals, with the significance testing based on the bootstrap inference. In that case, we apply the Cochrane–Orcutt-type transformation by applying the $(1 - L^{1} )$ transformation in succession to all the variables and repeating the estimation on the transformed variables. This is repeated until there is no significant autocorrelation left. Next, we eliminate any redundant variables and repeat the process. The result is that we are left with a model with no autocorrelation and a congruent selection of explanatory variables.

The essential point here is to estimate the autocorrelation coefficients from panel data. We apply a number of techniques, starting with the Han et al. (2014) HPS estimator based on the X-differencing approach. We also use the Han and Phillips (2010) HP estimator and the fixed-effects FE estimator (see, e.g. Baltagi 2013). To all these estimators, we apply corrections aimed at eliminating their bias, as proposed by Chudik et al. (2018) and Kao et al. (2021). Because autocorrelation and other types of weak dependency might still be present in the model, we base further inference on the moving block bootstrap (MBB) approach (see Gonçalves 2011; for its further development, see Qiu et al. 2019). The MBB estimates are robust to time dependence of unknown form, where the robustness does not depend on the assumption of normality.

Table 2 summarises some results obtained by applying this approach for the case where the widest version of OxCGRT is used, that is, GRI. We have 56 weekly observations in our data set for each country, from the first week of March 2020 until the fourth week of March 2021, which gives 392 data points for the seven countries. We show the results for five alternative model specifications in the columns marked [1], [2], [3], [4] and [5]. In these models, we use only one interaction effect for Ukraine with the stringency tracker. The data shown in Fig. 2 indicate that the relationship here might be different for other countries. This choice is confirmed further in our robustness analysis. The choice of which tests to apply has been determined by the panel of countries that differ substantially in their data reporting standards, social and political structure, media policy, degree of political freedom and centralisation and so forth. These might all affect the homogeneity of the panel.

**Table 2:** Estimation of selected models [1]—[5]. Dependent variable: weekly changes in the logarithms of pandemic-augmented and sentiment-weighted uncertainty index UC

	[1]	[2]	[3]	[4]	[5]
$g_{it - 1}$	-0.118	-0.127	-0.077	-0.076	-0.127
pval	0.002	0.001	0.047	0.048	0.001
MBBpval	0.002	0.010	0.104	0.116	0.003
$g_{it - 2}$			-0.042	-0.051	
pval			0.098	0.050	
MBBpval			0.069	0.047	
$\Delta g_{it - 1}$	0.042	0.051			0.052
pval	0.098	0.050			0.046
MBBpval	0.072	0.032			0.038
$new\_cases_{it - 1}$	0.027		0.027	0.010	
pval	0.147		0.147	0.405	
MBBpval	0.378		0.367	0.558	
$new\_deaths_{it - 1}$	0.576	0.010	0.576		
Pval	0.001	0.405	0.001		
MBBpval	0.012	0.603	0.014		
$UA \times g_{it - 1}$		0.598		0.598	0.600
Pval		0.000		0.000	0.000
MBBpval		0.005		0.014	0.008
$\rho_{1}$	0.354	0.352	0.354	0.352	0.352
bpval	0.000	0.000	0.000	0.000	0.000
$R^{2}$	0.058	0.056	0.058	0.056	0.056
Frees	-0.266	-0.216	-0.266	-0.216	-0.204
pval	0.395	0.415	0.395	0.415	0.419
Pesaran	0.617	0.620	0.617	0.620	0.634
pval	0.269	0.268	0.269	0.268	0.263
Baltagi-Li	-0.806	-0.812	-0.806	-0.812	-0.805
pval	0.210	0.208	0.210	0.208	0.210
Fixed Effects	2.294	2.568	2.294	2.568	2.592
pval	0.035	0.019	0.035	0.019	0.018

Estimation: Kao et al. (2021) fixed-effects method

$g_{it}$: *The Government Response Index*; $new\_cases_{it}$:logarithm of the number of new Covid-19 cases in country *i* during week *t*; $new\_deaths_{it}$: logarithms of the number of new Covid-19-related deaths in country *i* during week *t*; *UA*—dummy for Ukraine.

The symbols and abbreviations for the variables and statistical indicators are:

**Table:** 

$g_{it - k}$	Logarithms of the Government Response Index (GRI), lagged by $k$;
$\Delta g_{it - k}$	First differences of $g_{it - k}$;
$new\_cases_{it - k}$	Logarithms of the number of new Covid-19 cases registered during the week;
$new\_deaths_{it - k}$	Logarithms of the number of new Covid-19-related deaths during the week;
$UA \times g_{it - k}$	Interaction effect, where $UA = 1$ if an observation is identified as coming from Ukraine, and zero otherwise;
pval and MBBval	Analytical and moving block bootstrap (MBB) p-values;
$\rho_{k}$	Estimate of the $k$ th order autocorrelation coefficient;
bpval	Bootstrapped p-value for autocorrelation coefficients, where the MBB approach is not needed;
$R^{2}$	Coefficient of determination. If $\rho_{k} \ne 0$, $R^{2}$ is computed for Cochrane–Orcutt transformed variables;
Frees and Pesaran	Respectively Frees (1995) and Pesaran (2004) statistics for testing cross-sectional dependence in panel data models;
Baltagi-Li	Baltagi and Li (1995) statistic for testing serial correlation in panel data models;
Fixed Effects	F-statistic for testing the joint significance of countries’ fixed effects

As expected, the fixed effects in the model are significant. The autocorrelation and cross-dependence statistics are not significant, which somewhat unexpectedly confirms that the panel is statistically sound and the dynamics of the process are reasonably precisely modelled even though it describes text-based uncertainty in countries with vastly different economic, political and media control systems. The soundness of the panel is also confirmed by additional robustness analysis; see Table 5 and the discussion of it further on in this paper.

The estimates of the main parameters of interest, which are the variables representing the levels and changes of the government stringency measure, are quite stable across the specifications. Of particular note is that the estimates of the parameter on $g_{it - k}$ are significant and negative for all specifications. This confirms the conjecture that a higher level of government restrictions causes a reduction in uncertainty. The positive and significant parameter tells us that changing the restrictions leads to an increase in uncertainty, but the elasticity of this increase is smaller in absolute value than the elasticity of $g_{it - k}$. This is consistent with the expected hypothesised positive sign of $\mathop \sum \limits_{j = 1}^{n} \theta_{2,j}$ in (1).

The significance, and so the relevance, of the extraneous variables $new\_cases_{it - 1}$ and $new\_deaths_{it - 1}$ is not evident. Although the estimates for the parameters of these variables are positive for $new\_cases_{it - 1}$, as expected, the estimates are not significant in any of the three specifications shown for models [1], [3] and [4]. For $new\_deaths_{it - 1}$, they are not significant in model [2]. The congruent specification is [5] and adding $new\_deaths_{it - 1}$ to it and so extending from model [5] to [2] does not add much to the significance, and so we conclude that information about new cases of Covid and Covid-related deaths does not interfere in the relationship between the government’s anti-pandemic policy and uncertainty. Moreover, the parameters on $g_{it - 1}$ and $\Delta g_{it - 1}$ are virtually identical in models [2] and [4]. We conclude, therefore, that the congruent specification [5] is sufficient for investigating the relationship between a government’s anti-pandemic policy and uncertainty.

The high positive and significant interaction effect for Ukraine shows that a high level of government restrictions in this country actually increases uncertainty. A possible interpretation might be that Ukraine was a highly decentralised country with a high level of corruption in 2020 and 2021. A high level of restrictions might correspond to an increase in opportunities for corruption, which would then cause uncertainty to increase. This might also be an effect of the misinformation campaign, which distorted newspaper information and created mistrust in government policy (Patel et al. 2020).

Table 3 gives the results of the estimation of the congruent specification, marked by [5] in Table 1, using the five different estimation methods discussed briefly above and further referenced in the footnote to the table.

**Table 3:** Estimation of model [5], as in Table 2, by selected methods

	HPS	HPK	HPC	FEK	FEC
$g_{it - 1}$	-0.188	-0.134	-0.078	-0.127	-0.101
Pval	0.000	0.000	0.069	0.001	0.017
MBBpval	0.000	0.004	0.078	0.004	0.015
$\Delta g_{it - 1}$	0.140	0.060	0.016	0.052	0.030
Pval	0.000	0.025	0.319	0.046	0.175
MBBpval	0.001	0.020	0.256	0.057	0.118
$UA \times g_{it - 1}$	0.177	0.639	0.330	0.600	0.443
Pval	0.002	0.000	0.021	0.000	0.005
MBBpval	0.457	0.008	0.141	0.006	0.051
$\rho_{1}$		0.322	0.496	0.352	0.440
Bpval		0.000	0.000	0.000	0.000
$R^{2}$	0.282	0.067	0.017	0.056	0.029
Frees	0.085	-0.222	-0.034	-0.204	0.014
Pval	0.466	0.412	0.487	0.419	0.495
Pesaran	0.769	0.632	0.671	0.634	0.653
Pval	0.221	0.264	0.251	0.263	0.257
Baltagi-Li	6.215	-0.300	-2.944	-0.805	-2.171
Pval	0.000	0.382	0.002	0.210	0.015
Fixed Effects	7.657	3.015	0.838	2.592	1.420
Pval	0.000	0.007	0.541	0.018	0.206

HPS: Han, Phillips and Sul (2014) X-differences method with the Kao et al. (2021) correction; HPK: Han and Phillips (2010) HP method with the Kao et al. correction; HPC: HP method with the Chudik et al. (2018) correction; FEK: fixed-effects method with the Kao et al. (2021) correction; FEC: fixed-effects method with Chudik et al. (2018) correction

$g_{it}$: logarithm of *The Government Response Index*; *UA*—dummy for Ukraine.

It may be noted that the HPS method, which is the Han, Phillips and Sul (2014) X-differences method with the Kao et al. (2021) correction, collapses to a static fixed-effects method, as the estimates of the autocorrelation coefficients are insignificant. We confirm a very substantial bias of the autoregression coefficients given by this method using a limited Monte Carlo experiment, for which the results are available upon request. The method of Han and Phillips (2010) and the dynamic fixed effects, both with Kao et al. (2021) correction (columns marked HPK and FEK), are the most promising, as autocorrelation seems to be successfully removed here by the Cochrane–Orcutt procedure. The estimates of the parameters for these two methods are also quite close to each other.

We concentrate further on analysing the robustness of specification [5] estimated by the FE method with the Kao et al. correction, denoted FEK. For this model, we check how much the way we measure uncertainty affects the results. Table 4 shows the results of estimating the congruent model, where the dependent variable is (i) the first difference of the logarithms of U, that is $y_{it} = \Delta u_{it} = \Delta \log ({\text{U}}_{it} )$, where U is the uncertainty index not augmented by the pandemic-related elements and not weighted by sentiments; (ii) $y_{it} = \Delta u_{it}^{( - )} = \Delta \log ({\text{U}}_{it}^{( - )} )$ where ${\text{U}}^{( - )}$ is constructed as U, that is, without pandemic-related augmentation, but weighted by negative sentiments; and (iii) is our uncertainty measure ${\text{UC}}$, that is, $y_{it} = \Delta uc_{it} = \Delta \log ({\text{UC}}_{it} )$. The results given in Table 4 show that the government stringency measures do not affect the traditionally measured uncertainty based on the EPU descriptors, even if negative sentiments weigh the index. Although no autocorrelation is discovered in such models, the estimates are not statistically significant; they do, however, have the proper signs.

**Table 4:** Model [5] as of Table 2 with different dependent variables

	Dep. Var:$\Delta u_{it}$	Dep. Var: $\Delta u_{it}^{( - )}$-	Dep. Var:$\Delta uc_{it}$
$g_{it - 1}$	-0.011	-0.005	-0.127
pval	0.432	0.467	0.001
MBBpval	0.675	0.666	0.003
$\Delta g_{it - 1}$	0.079	0.089	0.052
pval	0.225	0.197	0.046
MBBpval	0.437	0.432	0.032
$UA \times g_{it - 1}$	0.083	0.091	0.600
pval	0.343	0.329	0.000
MBBpval	0.185	0.170	0.010
$\rho_{1}$			0.352
bpval			0.000
$R^{2}$	0.003	0.003	0.056
Frees	0.264	0.145	-0.204
pval	0.396	0.443	0.419
Pesaran	0.813	0.792	0.634
pval	0.208	0.214	0.263
Baltagi-Li	-0.530	-0.423	-0.805
pval	0.298	0.336	0.210
Fixed Effects	0.082	0.086	2.592
pval	0.998	0.998	0.018

*u*: logarithm of U, which is not augmented by pandemic-related elements and not weighted by sentiments; $u_{it}^{( - )}$: logarithm of ${\text{U}}^{( - )}$, which is the U index weighted by negative sentiments; $uc_{it}$: logarithm of pandemic-augmented and sentiment-weighted uncertainty index UC; $g_{it}$: *The Government Response Index*; *UA*—dummy for Ukraine.

We also conducted a detailed robustness analysis of the model. Firstly, we estimate the congruent model using alternative stringency trackers. We do this by constructing the $g_{it - 1}$ and $\Delta g_{it - 1}$ variables using, in turn, data on the Stringency Index (SI), the Legacy Stringency Index (LSI), the Containment and Health Index (CHI), and the Government Response Index (GRI), which we use in all previously discussed computations. These indices are explained in Sect. 2, and the estimation results are in Table 6 in Appendix B and summarised in the first three columns of Table 5. It shows that the widest index, GRI (containing all individual lockdown and restrictions measures), has the largest, in absolute values, coefficient, which indicates that all the individual measures might affect uncertainty. However, the relation between the inclusion of various measures into stringency and uncertainty is not linear. The weakest effect is shown by SI, which includes more individual measures than LSI.

**Table 5:** Selected results of the robustness test: Different stringency measures and exclusion of a country

Different stringency measures (in logarithms, $g_{it}$)			Exclusion of a country		
Stringency measure	Estimated parameter on $g_{it - 1}$	Estimated parameter on $\Delta g_{it - 1}$	Excluded country	$g_{it}$: logarithms of the Government Stringency Index, GRI	
				Estimated parameter on $g_{it - 1}$	Estimated parameter on $\Delta g_{it - 1}$
SI	–0.089	0.064	BR	–0.058	0.060
LSI	–0.121	0.069	KZ	–0.078	0.034
CHI	–0–127	0.052	PL	–0.088	0.048
GRI	–0.135	0.053	RU	–0.071	0.042
			UA	–0.137	0.072
			UK	–0.123	0.064
			US	–0.089	0.047
means	–0.118	0.060	means	–0.092	0.052
st.devs	0.017	0.007	st.devs	0.026	0.012

Dependent variable: weekly changes in the logarithms of pandemic-augmented and sentiment-weighted uncertainty index UC. Estimation method: fixed-effects method with Chudik et al. (2018) correction (FEK)

SI: The Stringency Index; LSI: The Legacy Stringency Index; CHI: The Containment and Health Index; GRI: The Government Response Index; BR: Belarus; KZ: Kazakhstan; PL: Poland; RU: Russia; UA: Ukraine; UK: United Kingdom; US: United States; means: the mean of the coefficients in the corresponding column; st.devs: standard division of the estimated parameters

Finally, we focus on the central issue of estimating the panel with data that describe vastly different countries. We conduct another robustness test by estimating the model with the data for one country removed so that we estimate it using data for six countries rather than seven, removing the data in turn for Belarus, Kazakhstan, Poland, Russia, the UK and the USA. The estimation results are given in Appendix B in Table 7 and summarised in the last three columns of Table 5 by showing the estimates of the parameters on $g_{it - 1}$ and $\Delta g_{it - 1}$ for a different choice of countries, with their means and standard deviations computed across the models. Not surprisingly, the biggest shift downwards in the effect of $g_{it - 1}$ on uncertainty is when Ukraine is excluded. A strong shift downwards is also visible when the UK is excluded. This might be because the UK has a reasonably high level of social conformity compared to the levels in the other countries in the panel. In this case, the formal introduction of the rules might not change uncertainty. The standard deviations are small, and the corresponding estimates in all these cases do not differ much from each other. This confirms that the results of our investigation are robust and interpretable, even though the dynamics of the process modelled might be explosive and unstable.

**Table 6:** Estimates of the congruent model using different government response stringency trackers

	Stringency index applied			
	SI	LSI	CHI	GRI
$g_{it - 1}$	-0.089	-0.121	-0.127	-0.135
pval	0.008	0.003	0.001	0.000
MBBpval	0.011	0.007	0.004	0.004
$\Delta g_{it - 1}$	0.064	0.069	0.052	0.053
pval	0.013	0.009	0.046	0.043
MBBpval	0.016	0.032	0.044	0.020
$UA \times g_{it - 1}$	0.341	0.426	0.600	0.574
pval	0.000	0.000	0.000	0.000
MBBpval	0.015	0.005	0.009	0.011
$\rho_{1}$	0.333	0.333	0.352	0.344
bpval	0.000	0.000	0.000	0.000
$R^{2}$	0.049	0.055	0.056	0.060
Frees	-0.126	-0.200	-0.204	-0.305
pval	0.450	0.421	0.419	0.380
Pesaran	0.695	0.672	0.634	0.630
pval	0.244	0.251	0.263	0.264
Baltagi-Li	-0.212	-0.223	-0.805	-0.687
pval	0.416	0.412	0.210	0.246
Fixed Effects	2.161	2.525	2.592	2.745
pval	0.046	0.021	0.018	0.013

Dependent variable: weekly changes in the logarithms of the pandemic-augmented and sentiment-weighted uncertainty index UC. Estimation method: fixed-effects method with Chudik et al. (2018) correction (FEK)

$g_{it}$: Logarithm of the corresponding stringency index.

SI: The Stringency Index; LSI: The Legacy Stringency Index; CHI: The Containment and Health Index; GRI: The Government Response Index; *UA*: dummy for Ukraine.

**Table 7:** Estimates of the congruent model with different countries excluded from panel

Excluded country	Belarus	Kazakhstan	Poland	Russia	Ukraine	UK	USA
$g_{it - 1}$	–0.058	–0.078	–0.088	–0.071	–0.137	–0.123	–0.089
pval	0.113	0.040	0.033	0.068	0.000	0.008	0.025
MBBpval	0.050	0.043	0.014	0.042	0.002	0.005	0.013
$\Delta g_{it - 1}$	0.060	0.034	0.048	0.042	0.072	0.064	0.047
pval	0.063	0.158	0.085	0.098	0.004	0.042	0.082
MBBpval	0.068	0.049	0.058	0.095	0.027	0.052	0.066
$\rho_{1}$	0.392	0.363	0.374	0.413	0.312	0.380	0.383
bpval	0.000	0.000	0.000	0.000	0.000	0.000	0.000
$R^{2}$	0.014	0.016	0.017	0.014	0.072	0.027	0.019
Frees	0.087	–0.221	–0.246	0.098	–0.266	–0.175	–0.286
pval	0.465	0.412	0.403	0.461	0.395	0.430	0.387
Pesaran	0.706	0.502	0.576	0.671	0.534	0.459	0.577
pval	0.240	0.308	0.282	0.251	0.297	0.323	0.282
Baltagi–Li	–0.631	–0.335	–0.519	–0.356	–0.986	–0.406	–0.544
pval	0.264	0.369	0.302	0.361	0.162	0.342	0.293
Fixed Effects	0.435	0.741	0.658	0.583	2.422	1.033	0.774
pval	0.824	0.593	0.655	0.713	0.036	0.398	0.569

Dependent variable: weekly changes in the logarithms of the pandemic-augmented and sentiment-weighted uncertainty index UC. Estimation method: fixed-effects method with Chudik et al. (2018) correction (FEK)

$g_{it - 1}$: Logarithm of The Government Response Index.

## Conclusions

Our results show that imposing and maintaining strong and consistent anti-pandemic policies not only reduced the spread of the virus, as has been widely documented elsewhere, but also managed to reduce economic uncertainty. At least, it did so in 2020 and early 2021 during the first waves of the pandemic, before the significant increase in the rate of vaccinations. The pre-emptive and long-lasting anti-pandemic policy decisions positively affected how pandemic-related economic uncertainty developed, as they reduced it and acted as a calming factor. This is evident for all the countries in our panel except Ukraine. However, inconsistencies in such policies that result in them being changed relatively frequently counteract these positive effects. The best way to reduce the economic uncertainty that arises from a pandemic and to flatten to some extent the waves of the pandemic would be to set the overall level of anti-pandemic restrictions at a reasonably high level relatively early and then refrain from changing those restrictions frequently. Another matter is whether such a policy would be politically, socially or economically acceptable. Refraining from changing the restrictions might also often prove unwise if there are no reliable models for forecasting the development of the pandemic and while understanding about the virus is developing rapidly.

Our econometric results are quite robust, notwithstanding possible bias in newspaper reporting on the pandemic, which might affect all the countries in the panel. The estimates of the parameters for policy are not affected much if different estimation methods are used, extraneous variables are excluded or included or countries are excluded from the panel. It should be stressed that excluding the USA and the UK, which are markedly different from the rest of the panel in numerous respects, does not affect the results in any significant way. Our results confirm that the dynamics of text-based uncertainty in response to anti-pandemic policy are fairly universal and are independent of the degree of press freedom, political constraints or media control and organisation. Nevertheless, interpretable and statistically viable results can only be obtained if the text-based measure of uncertainty is modified to account for appropriate pandemic-related words.

Our model can be improved and extended in the future. We can include data for subsequent waves of the pandemic, and we can also use richer data by including more newspapers, particularly for the UK and the USA. However, having more newspapers for those countries might be a lesser priority; as Fig. 1 suggests, for the USA at least, the results obtained using data from one major newspaper and from many newspapers might be similar.

Despite these shortcomings, we feel that our findings can be used to augment the earlier results about the possible path and speed of post-pandemic recovery obtained by Eichengreen et al. (2021), Ng (2020) and others. Government restrictions should be relaxed in a consistent and logical fashion based on forward-looking analysis to avoid a rise in pandemic-related uncertainty with all its negative consequences. Our results indirectly emphasise the need to set up anti-pandemic policies based on reliable forecasts rather than on the reported real-time data. In other words, it is better to set restrictions at a reasonably severe level and avoid frequent changes in them, which suggests an obvious analogy with the consistency principle of monetary policy.

## Data Availability

Available upon request.

## Appendix A

### Outline of the algorithm for constructing sentiment-weighted uncertainty indices

1. For each language, define sets of words related to economics, policy, uncertainty and sets of words describing the pandemic. These sets are called herein the descriptors and denoted, respectively, as {economic}, {policy}, {uncertainty} and {pandemic}.
2. Perform two types of searches, Search-1 and Search-2, of all the articles in the database (corpus), to identify those that contain at least one word from each set of descriptors. For Search-1, the set of descriptors is {economic}, {policy} and {uncertainty}. For Search-2, the set of descriptors is {economic}, {policy} and ({uncertainty} or {pandemic}).
3. Define two indicators for the newspaper article *i*, $I1$ and $I2$, as follows:
  $$I1(i) = I_{{\{ economic\} }} (i) \times I_{{\{ policy\} }} (i) \times I_{{\{ uncertainty\} }} (i)$$; and
  $I2(i) = I_{{\{ economic\} }} (i) \times I_{{\{ policy\} }} (i) \times I_{{\{ uncertainty\} \cup \{ pandemic\} }} (i),$ where $I_{{\{ \bullet \} }}$ is an indicator for a particular descriptor, that is, e.g. $I_{{\{ economic\} }} (i) = 1$ if article *i* contains at least one word from the descriptor {economic} and is zero otherwise.
  The way $I1$ is constructed is essentially the same as in the original Baker et al. (2016) EPU methodology. The method for $I2$ is similar, but not identical, to that of the Baker et al. (2020b) Covid-Induced Economic Uncertainty Index.
4. The article *i* is selected in Search-1 if $I1(i) = 1$, and in Search-2 if $I2(i) = 1$.
5. For each selected article *i,* that is where $I1(i) = 1$ or $I2(i) = 1$, compute fractions of words belonging to the lexicon of the corresponding language (see Sect. 2) of positive and negative words in the newspaper language.
6. Divide the set of fractions computed for all articles of each newspaper into five groups, decided by the quantiles of the distribution of the fractions. That is, group 1 contains all articles where the frequency of words belonging to the lexicon is below the 0.15th quantile, and, similarly, group 2 includes articles between the 0.15 h and 0.50th quantile, group 3 between 0.5th and 0.75th quantile, group 4 between 0.75th and 0.90th quantile and group 5 above the 90th quantile.
7. On this basis, construct sentiment measure $S_{{}}^{ + } (\cdot)$ for positive sentiments such that $S_{{}}^{ + } (i) = 0.15$ for article *i*, where the fraction of positive words in the distribution is smaller than 0.15; $S_{{}}^{ + } (i) = 0.50$ where this fraction is between 0.15 and 0.50, etc. Analogously, we constructed the measure $S_{{}}^{ - } (\cdot)$ for negative sentiments. For the description of the methodology scaling, see Ferrara and Yang (2015); see also Thelwall et al. (2010). We have also applied a variation of this methodology, where we constructed the distribution of fractions jointly for all articles for countries with more than one newspaper in the corpora. The results are very similar to those presented here.
8. Weight indicators $I1$ and $I2$ for each article *i* by its corresponding sentiment measures $S_{i}^{ + }$ and $S_{i}^{ - }$ in the following way:
  $$I1^{sign} (i) = I1(i) \times \omega_{i}^{{{\text{s}} ign}}$$, and $$I2^{sign} (i) = I2(i) \times \omega_{i}^{{{\text{s}} ign}}$$,
  where $$sign \in \{ - , + \}$$, and $$\omega_{i}^{sign} = 1 + S_{i}^{sign}$$.
9. For computing index not weighted by the sentiments, that is for index denoted by ${\text{U}}$ in the main text, aggregate and standardise all selected $I1$’s as in the Baker et al. 2016, EPU index. For computing $U^{sign}$ and $UC$, do the analogous aggregation, but using $I1^{sign}$ and $I2^{ - }$ correspondingly.

Note that it is also plausible to compute indices weighted by the balance of sentiments, $I_{i}^{ \pm } = I_{i} \times \omega_{i}^{ \pm }$, where $\omega_{i}^{ \pm } = 1 - (S_{i}^{ + } - S_{i}^{ - } )$. In this paper, we present results obtained with the use of the indices weighted by negative sentiments. Conclusions obtained with the use of positive sentiments and balance of sentiments are analogous to these presented here.

### Appendix B: Additional results for the robustness analysis

See Tables 6, 7.

## References

[CR1] Abazi V (2020). ‘Truth distancing? Whistleblowing as remedy to censorship during COVID-19. Eur J Risk Regulation.

[CR2] Ahir H, Bloom N, Furceri B (2021) World pandemic uncertainty index [WUPI]. FRED, Federal Reserve Bank of St. Louis; https://fred.stlouisfed.org/series/WUPI

[CR3] Altig D, Baker S, Barrero JM, Bloom N, Bunn P, Chen S, Davis SJ, Leather J, Meyer B, Mihaylov E, Mizen P, Parker N, Renault T, Smietanka P, Thwaites G (2020). Economic uncertainty before and during the Covid-19 pandemic. J Public Econ.

[CR4] Angelone E (2010) Uncertainty, uncertainty management and metacognitive problem solving in the translation task, in (G.M. Shreve and E. Angelone, eds.) *Translation and cognition*, John Benjamins, Amsterdam, pp 17–40

[CR5] Apaitan T, Luangaram P, Manopimoke P (2022). Uncertainty in an emerging market economy: evidence from Thailand. Emp Econ.

[CR6] Arnon A, Ricco J, Smetters K (2020) Epidemiological and economic effects of lockdown. Brookings Papers on Economic Activity BPEA Conference Drafts

[CR7] Babrow AS (2001). Uncertainty, value, communication and problematic integration. J Commun.

[CR8] Bagus P, Peña-Ramos JA, Bayón AS (2021). COVID-19 and the political economy of mass hysteria. Int J Environ Res Public Health.

[CR9] Baig A, Butt HA, Haroon O, Rizvi SAR (2021). Deaths, panic, lockdowns and US equity markets: The case of COVID-19 pandemic. Financ Res Lett.

[CR10] Baker SR, Bloom N, Davis SJ (2016). Measuring economic policy uncertainty. Quart J Econ.

[CR11] Baker SR, Bloom N, Davis SJ, Kost KJ, Sammon MC, Viratyosin T (2020) The unprecedented stock market impact of COVID-19, NBER Working Paper 26945

[CR12] Baltagi BH (2013). Econometric analysis of panel data.

[CR13] Baltagi BH, Li Q (1995). Testing AR(1) against MA(1) disturbances in an error component model. J Econ.

[CR14] Bargain O, Aminjonov U (2020). Trust and compliance to public health policies in times of COVID-19. J Public Econ.

[CR15] Barrero JM, Bloom N (2020) Economic uncertainty and the recovery. https://www.kansascityfed.org/documents/7115/BloomPaper_JH2020.pdf.

[CR16] Berman N, Couttenier M, Monnet N, Ticku R (2022). Shutdown policies and conflict worldwide. J Comp Econ.

[CR17] Blei DM, Ng AY, Jordan MI (2003). Latent Dirichlet allocation. J Mach Learn Res.

[CR18] Bloom N, Floetotto M, Jaimovich N, Saporta-Eksten I, Terry SJ (2018). Really uncertain business cycles. Econometrica.

[CR19] Brodeur A, Clark AE, Fleche S, Powdthavee N (2021). COVID-19, lockdowns and well-being: evidence from Google Trends. J Public Econ.

[CR20] Campos J, Ericsson NR, Hendry DF (2005) General-to-specific modelling: an overview and selected bibliography. Board of Governors of the Federal Reserve System, International Finance Discussion Papers 838

[CR21] Charemza W, Makarova S, Rybiński K (2022). Economic uncertainty and natural language processing; the case of Russia. Economic Analysis and Policy.

[CR22] Chudik A, Pesaran HM, Yang J-C (2018). Half-panel jackknife fixed-effects estimation of linear panels with weakly exogenous regressors. J Appl Economet.

[CR23] Clarke JN, Everest MM (2006). Cancer in the mass print media: fear, uncertainty and the medical model. Soc Sci Med.

[CR24] Cochrane D, Orcutt GH (1949). Application of least squares regression to relationships containing auto-correlated error terms. J Am Stat Assoc.

[CR25] Coibion O, Gorodnichenko Y, Weber M (2020) The cost of the Covid-19 crisis: lockdowns, macroeconomic expectations and consumer spending. NBER Working Paper 27141

[CR26] Cot C, Cacciapaglia G, Sannino F (2021). Mining Google and Apple mobility data: temporal anatomy for COVID-19 social distancing. Sci Rep.

[CR27] Devlin J, Chang MW, Lee K, Toutanova K (2019) BERT: pre-training of deep bidirectional transformers for language understanding. In: Proceedings of 7th annual conference of the North American chapter of the association for computational linguistics: human language technologies, pp 4171–4186. 10.48550/arXiv.1810.04805

[CR28] Dyer O (2020) Covid-19: Russia admits to understating deaths by more than two thirds. BMJ 371. 10.1136/bmj.m4975 10.1136/bmj.m4975 33384283

[CR29] Eichenbaum MS, Rebelo S, Trabandt M (2021) The macroeconomics of epidemics. NBER Working Papers 26882

[CR30] Eichengreen B, Park D, Shin K (2021). The shape of recovery: Implications of past experience for the duration of the COVID-19 recession. J Macroecon.

[CR31] Ferrara E, Yang Z (2015). Measuring emotional contagion in social media. PLoS ONE.

[CR32] Frees EW (1995). Assessing cross-sectional correlation in panel data. J Economet.

[CR33] Fu Y, Dhonnchadha EU (2020) A pattern-mining driven study on differences of newspapers in expressing temporal information. . 10.48550/arXiv.2011.12265

[CR34] Gonçalves S (2011). The moving blocks bootstrap for panel linear regression models with individual fixed effects. Economet Theor.

[CR35] Gros D, Ounnas A, Yeung TY-C (2021). A new Covid policy stringency index for Europe. Covid Econ Vetted Real Time Papers.

[CR36] Hale T, Angrist N, Goldszmit R, Kira B, Petherick A, Phillips T, Webster S, Cameron-Blake E, Hallas L, Majumdar S, Tatlow H (2021). A global panel database of pandemic policies (Oxford COVID-19 Government Response Tracker). Nat Human Behav.

[CR37] Han C, Phillips PCB (2010). GMM estimation for dynamic panels with fixed effects and strong instruments at unity. Economet Theor.

[CR38] Han C, Phillips PCB, Sul D (2014) X-differencing and dynamic panel model estimation. Economet Theory 30:201–251. 10.1017/S0266466613000170

[CR39] Jha M, Liu H, Manela A (2021) Does finance benefit society? A language embedding approach. In: Presented at 2nd workshop ‘big data and economic forecasting’. European Commission

[CR40] Kao C, Liu L, Sun R (2021). A bias-corrected fixed effects estimator in the dynamic panel data model. Emp Econ.

[CR41] Kim W (2021). Government spending policy uncertainty and economic activity: US time series evidence. J Macroecon.

[CR42] Kotelnikova A, Bochenina K, Kotelnikov E (2021). Lexicon-based methods vs. BERT Text Sentiment Anal.

[CR43] Liu T, Nakajima T, Hamori S (2022). The impact of economic uncertainty caused by COVID-19 on renewable energy stocks. Emp Econ.

[CR44] Loukachevitch N, Levchik A (2016) Creating a general Russian sentiment lexicon’. In: Proceedings of language resources and evaluation conference LREC-2016, pp 1171–1176

[CR45] McMahon M (2019) The macroeconomics of uncertainty. In: Troeger VE (ed) Which way now? Economic policy after a decade of upheaval A CAGE Policy Report, The Social Market Foundation

[CR46] Mdaghri AA, Abdessamad R, Raghibi A, Thanh CN, Oubdi L (2021). Stock market liquidity, the great lockdown and the COVID-19 global pandemic nexus in MENA countries. Rev Behav Finance.

[CR47] Meyer B, Mihaylov E, Davis SJ, Parker N, Altig D, Barrero JM, Bloom N (2021) Pandemic era uncertainty on main street and wall street. Federal Reserve Bank of Atlanta, Working Paper 2021–2. https://www.frbatlanta.org/-/media/documents/research/publications/wp/2021/01/15/02-pandemic-era-uncertainty-on-main-street-wall-street.pdf

[CR48] Mumtaz H, Theodoridis K (2017). Common and country specific economic uncertainty’. J Int Econ.

[CR49] Nalban V, Smădu, and A. (2021). Asymmetric effects of uncertainty shocks: Normal times and financial disruptions are different. J Macroecon.

[CR50] Nemira A, Ezekiel Adeniyi A, Gasich EL, Bulda KY, Valentovich LN, Krasko AG, Glebova O, Kirpich A, Skums P (2021) SARS-CoV-2 transmission dynamics in Belarus revealed by genomic and incidence data analysis. medRxiv. 10.1101/2021.04.13.21255404 10.1038/s43856-021-00031-1 PMC9053244 35602211

[CR51] Patel SS, Moncayo OE, Conroy KM, Jordan D, Erickson TB (2020) The landscape of disinformation on health crisis communication during the COVID-19 pandemic in Ukraine: hybrid warfare tactics, fake media news and review of evidence. JCOM-Masterfile-preprint, https://nrs.harvard.edu/URN-3:HUL.INSTREPOS:37364388.10.22323/2.19050202 PMC8425291 34504624

[CR52] Pesaran MH (2004) General diagnostic tests for cross section dependence in panels. University of Cambridge, Faculty of Economics, Cambridge Working Papers in Economics No. 0435

[CR53] Phillips PCB, Magdalinos T (2007). Limit theory for moderate deviations from a unit root. J Economet.

[CR54] Pulejo M, Querubín P (2021). Electoral concerns reduce restrictive measures during the COVID-19 pandemic. J Public Econ.

[CR55] Qiu J, Ma Q, Wu L (2019). A moving blocks empirical likelihood method for panel linear fixed effects models with serial correlations and cross-sectional dependences. Econ Model.

[CR56] Redl C (2020). Uncertainty matters: Evidence from close elections. J Int Econ.

[CR57] Ribeiro B, Hartley S, Nerlich B, Jaspal R (2018). Media coverage of the Zika crisis in Brazil: The construction of a ‘war’ frame that masked social and gender inequalities. Soc Sci Med.

[CR58] Speier M (2021) COVID-19 and the threat to press freedom in Central and Eastern Europe. Council on Foreign Relations 6, https://www.cfr.org/in-brief/covid-19-and-threat-press-freedom-central-and-eastern-europe

[CR59] Taboada M, Brooke J, Tofiloski M, Voll K, Stede M (2011). Lexicon-based methods for sentiment analysis. Comput Linguist.

[CR60] Tao Y, Yu J (2020). Model selection for explosive models. In: Essays in Honor of Cheng Hsiao. Adv Economet.

[CR61] Thelwall M, Buckley K, Paltoglou G, Cai D, Kappas A (2010). Sentiment strength detection in short informal text. J Am Soc Inform Sci Technol.

[CR62] Thomson EA, White PRR, Kitley P (2008). “Objectivity” and “hard news” reporting across cultures. J Stud.

[CR63] Yadav K, Erdoğdu U, Siwakoti S, Shapiro JN, Wanless A (2021). Countries have more than 100 laws on the books to combat misinformation. How well do they work?. Bull Atomic Sci.

[CR64] Yang S, Zhang W, Yuan Z (2021). Media reports of the COVID-19 pandemic: a computational text analysis of English reports in China, the UK, and the US. Adv J Commun.

[CR65] Yegorov S, Goremykina M, Ivanova R, Good SV, Babenko D, Shevtsov A, on behalf of the Semey COVID-19 Epidemiology Research Group, (2021) Epidemiology, clinical characteristics, and virologic features of COVID-19 patients in Kazakhstan: A nation-wide retrospective cohort study. The Lancet Regional Health – Europe 4, 100096. 10.1016/j.lanepe.2021.100096 10.1016/j.lanepe.2021.100096 PMC8050615 33880458

[CR66] Zaśko-Zielińska M, Piasecki M, Szpakowicz S (2015) A large Wordnet-based sentiment lexicon for Polish. In: Proceedings of the international conference recent advances in natural language processing (RANLP’2015), p 721–730

[CR67] Zhang W, Hamori S (2021a) The connectedness between the sentiment index and stock return volatility under COVID-19. A time-varying parameter vector autoregression approach. The Singapore Economic Review. 10.1142/S0217590822500023

[CR68] Zhang Y, Hamori S (2021). Do news sentiment and the economic uncertainty caused by public health events impact macroeconomic indicators? Evidence from a TVP-VAR Decomposition Approach. Q Rev Econ Finance.

[CR69] Zhao E, Wu Q, Crimmins EM, Ailshire JA (2020). Media trust and infection mitigating behaviours during the COVID-19 pandemic in the US. BMJ Glob Health.

